# Exogenous L-Arginine Enhances Pathogenicity of *Alternaria alternata* on Kiwifruit by Regulating Metabolisms of Nitric Oxide, Polyamines, Reactive Oxygen Species (ROS), and Cell Wall Modification

**DOI:** 10.3390/jof10110801

**Published:** 2024-11-19

**Authors:** Di Wang, Lingkui Meng, Haijue Zhang, Rong Liu, Yuhan Zhu, Xinyu Tan, Yan Wu, Qingchao Gao, Xueyan Ren, Qingjun Kong

**Affiliations:** Xi’an Key Laboratory of Characteristic Fruit Storage and Preservation, Shaanxi Engineering Laboratory of Food Green Processing and Safety Control, College of Food Engineering and Nutritional Science, Shaanxi Normal University, Xi’an 710119, China; wangdi237@snnu.edu.cn (D.W.);

**Keywords:** L-arginine, *Alternaria alternata*, signal transduction, pathogenicity, ROS metabolism, cell degradation

## Abstract

Black spot, one of the major diseases of kiwifruit, is caused by *Alternaria alternata*. A comprehensive investigation into its pathogenicity mechanism is imperative in order to propose a targeted and effective control strategy. The effect of L-arginine on the pathogenicity of *A. alternata* and the underlying mechanisms were investigated. The results showed that treatment with 5 mM L^−1^ of L-arginine promoted spore germination and increased the colony diameter and lesion diameter of *A. alternata* in vivo and in vitro, which were 23.1% and 9.3% higher than that of the control, respectively. Exogenous L-arginine treatment also induced endogenous L-arginine and nitric oxide (NO) accumulation by activating nitric oxide synthase (NOS), arginine decarboxylase (ADC) and ornithine decarboxylase (ODC). In addition, exogenous L-arginine triggered an increase in reactive oxygen species (ROS) levels by activating the activity and inducing gene expression upregulation of NADPH oxidase. The hydrogen peroxide (H_2_O_2_) and superoxide anion (O_2_^.−^) levels were 15.9% and 2.2 times higher, respectively, than in the control group on the second day of L-arginine treatment. Meanwhile, antioxidant enzyme activities and gene expression levels were enhanced, including superoxide dismutase (SOD), catalase (CAT), ascorbate peroxidase (APX), glutathione peroxidase (GPX), and glutathione reductase (GR). In addition, exogenous L-arginine stimulated cell wall-degrading enzymes in vivo and in vitro by activating gene expression. These results suggested that exogenous L-arginine promoted the pathogenicity of *A. alternata* by inducing the accumulation of polyamines, NO, and ROS, and by activating systems of antioxidants and cell wall-degrading enzymes. The present study not only revealed the mechanism by which low concentrations of L-arginine increase the pathogenicity of *A. alternata*, but also provided a theoretical basis for the exclusive and precise targeting of *A. alternata* in kiwifruit.

## 1. Introduction

Fresh fruits and vegetables are highly susceptible to infection by pathogenic fungi during post-harvest storage and transportation, resulting in decay that seriously affects quality [1]. *Alternaria alternata* is one of the most common rot-causing fungi of fruits and vegetables, and can cause black spot disease on a variety of fruits and vegetables, such as apples, kiwifruits, grapes, peaches, etc., resulting in serious postharvest losses [2]. In addition, the toxins produced by *A. alternata*, including tenuazonic acid (TeA), alternariol (AOH), and alternariol monomethyl ether (AME), are harmful to human health [2]. To solve these problems, many studies have focused on the inhibitory effect of *A. alternata*, while few studies have been conducted on the pathogenicity in infection. Therefore, it is necessary to study the pathogenicity mechanism of *A. alternata* and provide a theoretical basis for its precise control strategy.

L-arginine is a semi-essential amino acid that plays an important role in regulating protein synthesis and substance metabolism of fungi. Aron et al. [3] showed that, in *Magnaporthe oryz,a* hyphal growth and appressorium formation are affected by impaired arginine synthesis. Namiki et al. [4] demonstrated that arginine auxotrophic mutations resulted in reduced pathogenicity in *Fusarium oxysporum* f. sp. Melonis. Moreover, the metabolic products of L-Arginine, such as NO andpolyamine, play important roles in regulating growth and development, body metabolism, and signal transduction [5]. Gong et al. [6] demonstrated that L-arginine is required for the sporulation of shield *Coniothyrium minitans*, and its derivative NO may mediate its sporulation function.

Polyamines (PAs) are small, positively charged, bioactive molecules that are present in all living organisms [7]. The PAs in fungi include putrescine (Put), spermidine (Spd) and spermidine (Spm) [8,9,10]. Recent studies have shown that polyamines play an important role in the growth and development of pathogenic fungi, the resistance to oxidative stress of the host, and the synthesis of pathogenic. In *M. oryzae*, PAs promote the formation of appressorium infection structures and the expansion of rice cuticle cells [11]. Cheng et al. [12] showed that PAs stimulate germination and hyphal branching in the early stage of *Glomusetunicatum colonizatio*. Valdes-Santiago et al. [13] showed that PAs protect *Ustilago maydis* from salt and osmotic stress and influence their virulence performance. In *Ralstonia solanacearum*, putrescine is thought to be the toxic metabolites produced by pathogens [14]_._

Nitric oxide (NO) is an important signaling molecule, which is involved in a series of key signaling pathways and regulates a variety of physiological processes. NO is mainly derived from nitric oxide synthase-like (NOS-like) in fungi. In the fungal cytoplasm, NOS-like converts arginine to citrulline, resulting in the production of NO [15]. It has been reported that NO affects the spore production of *Puccinia striiformis* [16], the growth and development in *Stemphylium eturmiunum* [17], and the appressorium formation in rice blast fungus [18]. Additionally, NO was also found to regulate spore formation and germination in *B. emersonii*, and *Schizosaccharomyces* pombe by mediating the cGMP signaling pathway [19]. In *P. striiformis*, endogenous NO can also regulate fungal growth and development by regulating the level of reactive oxygen species (ROS) [20].

During plant infections, both pathogens and plants experience a burst of reactive oxygen species (ROS) [21]. In fungi, hydrogen peroxide (H_2_O_2_) and superoxide (O_2_^.−^) are common ROS, and ROS are mainly produced by reduced nicotinamide adenine dinucleotide phosphate oxidase (NADPH oxidase (NOX)) as it transfers electrons from NADPH to molecular oxygen [21]. As a key signaling molecule, ROS has a dual role for plants, animals and microorganisms. Excessive ROS cause oxidative damage to proteins, deoxyribonucleic acid (DNA), and lipids in organisms. The inhibitory effect of high levels of ROS has been demonstrated in *Magnaporthe grise* [22], *Aspergillus flavus* [23], *Aspergillus ochraceus* [23], *Fusarium oxysporum* [24], and *Botrytis cinerea* [24]. But, appropriate concentrations of ROS are important for the physiological activity of the pathogen, including mycelial growth, conidial differentiation, and the formation of substrate infestation structures [25]. It has been proved that low levels of ROS are necessary for appressorium formation during the infection of *Puccinia triticina* [25], *and* rice blast fungus [26], which is also essential for the development of the infection process in *pathogens*. Accordingly, the production or scavenging of ROS at specific stages during pathogen infection is also critical [27]. The reactive oxygen scavenging system of the pathogen, including antioxidant enzymes such as superoxide dismutase (SOD), catalase (CAT), ascorbate peroxidase (APX), glutathione peroxidase (GPX), and glutathione reductase (GR), which are activated during the infection process to reduce the excess ROS produced in pathogens intracellularly and in plant tissues, ultimately prevent oxidative damage to cells [27]. Zhang et al. [28] found that the knockout of the ROS-producing gene NOXR resulted in decreased levels of O_2_^.−^ and H_2_O_2_ in the mycelium, with a consequent decrease in SOD and CAT activities. Studies reported that exogenous H_2_O_2_ induced endogenous ROS accumulation in *A. alternata*, accompanied by an increase in SOD, CAT, and APX activities in response to oxidative stress [29]. Antioxidant genes were found to be up-regulated after the successful colonization of plant roots by *Arbuscular Mycorrhizae* [30]. Moreover, the loss of SOD1 activity in *Oidiodendron maius* increased the sensitivity of the fungus to ROS [31]. All the above studies have highlighted the importance of the ROS scavenging system for pathogen infection and pathogenicity.

Plant cell wall components are composed of cellulose, pectin. and hemicellulose, which act as a protective barrier against the pathogen infection [32]. In response, pathogens synthesize cell wall-degrading enzymes (CWDEs) during the infection process, which damage plant tissues and accelerate pathogen infection [32]. Pathogens secrete different types of CWDEs and it is generally believed that the pathogenicity of pathogenicity is closely related to the activity of CWDEs [32]. Common cell wall-degrading enzymes in fungi include cellulase (Cx), β-1,3-glucanase, polygalacturonase (PG), pectin methylesterase (PME), pectin methylgalacturonase (PMG), polygalacturonate trans-eliminating enzyme (PGTE), and pectin methyl trans-eliminating enzyme (PMTE) [33]. PMG, PG, Cx and β-glucosidase secretion have been detected in *Rhizoctonia solani* in vitro and in tobacco tissues, which promoted the development of the infection process of the pathogen [34]. *Fusarium equiseti* was found to secrete PG and cause fusarium wilt when infecting pitaya fruit, and the PG gene knockout mutants showed reduced pathogenicity [35]. Therefore, CWDEs secreted by a pathogen might play a pathogenic role as virulence factors during infection of the host plant.

Currently, most research has focused on the inhibition of *A. alternata* and the induction of host resistance to L-arginine. Few studies have investigated the effect of L-arginine on the pathogenicity of *A. alternata.* To better understand the role of exogenous L-arginine, different concentrations of L-arginine were used to investigate the effect of exogenous L-arginine on endogenous L-arginine, polyamines, NO, ROS, and cell wall degradation pathways to clarify the potential regulatory mechanisms of L-arginine in the pathogenicity of *A. alternata*.

## 2. Materials and Methods

### 2.1. Chemicals and Reagents

L-arginine (99%, AR) was purchased from Aladdin Reagent (Shanghai, China). Standards of ergosterol (≥98%), and high efficiency liquid chromatography (HPLC) grade methanol (≥99%) were purchased from Sigma Chemical Co. (St Louis, MO, USA). All the reagents were of analytical grade and were purchased from Shanghai Macklin Biochemical Co., Ltd. (Shanghai, China).

### 2.2. Kiwifruit and Pathogen Treatment and Storage

*A. alternata* was obtained from the College of Food Science and Engineering, Gansu Agricultural University, China. *A. alternata* was subcultured on potato dextrose agar (PDA) at 25 °C for 7 days, and the conidial suspension (10^6^ spores × 10^−3^ L^−1^) was prepared for inoculation.

‘Cuixiang’ kiwifruit (*Actinidia delicious cv. Cuixiang*) (total soluble solids content of 6.5% ± 1.3%) with uniform size and without injury and disease were selected from a market in town of Zhouzhi, Shaanxi Province, China, and immediately transported to the laboratory. The fruits were immersed in sodium hypochlorite and washed with sterile water. After air drying, the fruits were used for inoculation.

### 2.3. Spore Germination Assay

The assay of spore germination was based on the method of Li et al. [2] with minor modifications. L-arginine solution at concentrations of 0, 2.5, 5.0, 7.5 and 10 mM L^−1^ was added to the surface of a sterile water agar cake (8 mm in diameter), and then 10 μL of spore suspension (1 × 10^6^ spores mL^−1^) continued to be dropped on the surface of sterile water agar, respectively. Petri dishes containing spores were incubated at room temperature for 12 h. The rate of spore germination was determined every 2 h at 25 °C under a light microscope every 2 h. The spore germination rate was determined every 2 h under a light microscope (Olympus, Tokyo, Japan). Approximately 200 spores were selected and counted using a haemocyte counting plate to calculate the spore germination rate. Three replicates were used for each group.

### 2.4. In Vitro Mycelial Growth and In Vivo Pathogenicy Assessment of A. alternata

The assessment of in vitro mycelial growth and in vivo pathogenicity was based on the method of Li et al. [2] with minor modifications. *A. alternata* was cultured on potato dextrose agar (PDA) for seven days, and the spore suspension (10^6^ spores × 10^−3^ L^−1^) was prepared for inoculation. A total of 300 kiwifruits were selected and randomly divided into two groups of 150 each (control group and elicitor-treated groups), with 3 replicates of 50 each. The kiwifruits were surface-sterilized with 75% alcohol, and spread out on the test bench to allow the surface moisture to dry naturally, and then uniformly punched on the surface of the tubers with a perforator (4 mm in diameter). The holes were inoculated with 20 μL of spore suspension and 5 μL of different concentrations of L-arginine (0, 2.5, 5.0, 7.5, 10 mM L^−1^), then dried and stored at 25 (±2 °C). The wounded inoculated fruits were stored at 22 °C, and the diameter of the black spot lesions was determined by the criss-cross method after 16 days.

Agar disks containing *A. alternata* mycelium were taken using a punch and inoculated in the centre of PDA plates. Sterile water and 2.5, 5.0, 7.5, 10 mM concentrations of the L-arginine solution were then added to the surface of the medium, respectively. The culture was then incubated at 22 °C for 5 d. Mycelial growth was observed and the diameter of the *A. alternata* in vitro was determined after 5 d using the criss-cross method. A further 5.0 mM L-arginine was selected as the best from the preliminary experiments.

### 2.5. Measurement of Electrolyte Leakage, Ergosterol Content, and Malondialdehyde (MDA) Content

Electrolyte leakage, and MDA content were measured according to Li et al. [36]. *A. alternata* was incubated on PDA medium for 7 d at 28 °C in the dark, and then agar disks (6 mm diameter) containing *A. alternata* mycelium were taken with a punch. The agar disks containing *A. alternata* mycelium were inoculated into 250 mL of PDB medium (containing 0, 2.5, 5.0, 7.5, and 10 mM L-arginine, respectively) and incubated at 25 °C at 150 rpm min^−1^ with constant temperature in a shaking incubator (Shanghai Fuma experimental Equipment Co., Ltd., Shanghai, China). Mycelium samples were collected on days 0, 1, 2, 3, 4 and 5. The samples were frozen with liquid nitrogen and stored at −80 °C in the refrigerator (Panasonic Co., Ltd., Osaka, Japan) for subsequent physiological and biochemical analysis.

#### 2.5.1. Electrolyte Leakage

Ultrapure water (20 mL) was added to 6 agar disks containing *A. alternata* mycelium, and electrolyte leakage from the mycelium was detected using a conductivity meter (Model 105Aplus, Thermo Fisher Scientific, Sunnyvale, CA, USA). After boiling for 5 min, the electrolyte leakage was measured again.

The electrolyte leakage (%) = C_1_ − C_w1_/C_2_ − C_w2_%.

where C_1_ and C_2_ represented the electrolyte leakage of the sample before and after boiling; and C_w1_ and C_w2_ represented the electrolyte leakage of ultrapure water before and after boiling, respectively.

#### 2.5.2. Ergosterol Content

The mycelium was washed three times with 25 mM PBS buffer (pH 7.4) and collected on round filter paper (12 cm diameter) (Hangzhou Special paper Co., Ltd., Hangzhou, China). Mycelium tissue (0.5 g) was mixed with 2.5 mL of PBS buffer and 6 mL of saponifier (90% ethanol solution containing 15% NaOH) and saponified in a water bath at 80 °C for 60 min and cooled to room temperature. The mixture was extracted three times with 6 mL of petroleum ether (boiling range 30~60 °C), and dried in a water bath at 60 °C after two washes with 6 mL of distilled water. The mixture was then dissolved in cyclohexane and was adjusted to 1 mL g^−1^ mycelium. The determination of ergosterol content was performed using a HPLC system (Agilent Technologies Co, Santa Clara, CA, USA) equipped with a C_18_ column (250 mm × 4.6 mm, 5 μm). High-purity methanol was used as the mobile phase, and the injection volume of 20 μL was added at a flow rate of 1.0 mL min^−1^. The detection wavelength was 282 nm and the ergosterol content was expressed as mg g^−1^ on a dry weight (DW) basis.

#### 2.5.3. MDA Content

A total of 1 g of frozen mycelium was added to 2 mL of 150 mM phosphate buffer (pH 7.8) and ground in an ice bath. The mixture was then transferred to a 10 mL centrifuge tube and centrifuged at 4 °C for 20 min at 12,000 rpm to collect the supernatant for the MDA content assay.

An MDA content assay was performed using a malondialdehyde (MDA) assay kit (BC0020, Solarbio, Beijing, China) following the manufacture’s instruction. The measured MDA content of the *A. alternata* samples was expressed as mmol mg^−1^ protein.

### 2.6. Analyzation of Contents Related Substances and Enzyme Activities

#### 2.6.1. Mycelium Culture

*A. alternata* was incubated on PDA medium for 7 d in a biochemical incubator (MJP 250, Shanghai Keheng Industrial Development Co., Ltd., Shanghai, China) at 28 °C in the dark, and then agar disks (6 mm diameter) containing *A. alternata* mycelium were taken with a punch. The agar disks containing *A. alternata* mycelium were inoculated into 250 mL of PDB medium (containing 0 mM and 5.0 mM L-arginine, respectively) and incubated at 25 °C at 150 rpm min^−1^ with a constant temperature in a shaking incubator (QYC-2102C, Shanghai Fuma experimental Equipment Co., Ltd., Shanghai, China). The mycelium was frozen using liquid nitrogen, and stored at −80 °C in the refrigerator (Panasonic Co., Ltd., Osaka, Japan) for further analysis of the content-related substances and enzyme activities.

#### 2.6.2. Measurement of Arginine, Put, Spd, and Spm

Arginine was extracted by the method of Micallef et al. [37] with slight modification. Two mycelia were taken, homogenized by adding 4 mL of 3% (*w*/*v*) 5-sulfosalicylic acid solution, centrifuged (FRESCO21, Thermo Fisher Scientific, Sunnyvale, CA, USA) at 12,000× *g* for 10 min at 4 °C, and the supernatant was adjusted to pH 7 by using 4 mol·L^−1^ NaOH solution, and then filtered through a 0.45 μm membrane filter. The filtrate was collected and derivatized according to the instructions of the Waters AccQ·FluorTM Amino Acid Derivatization Kit. Arginine was analyzed by high performance liquid chromatography (HPLC). The HPLC was equipped with a 4 μm column and a diode array detector (DAD) for amino acids. The mobile phase consisted of acetonitrile (solvent A), ultrapure water (solvent B) and buffer (solvent C), and the elution gradients were as follows: 0 min, 100% A; 0.5 min, 99% A∶1% B; 18 min, 95%A∶5% B; 19 min, 91% A∶9% B; 29.5, min 83% A∶17% B; 33 min, 60% B∶40% C; 36 min, 100% A; 65 min, 60% B∶40% C; 36 min, 100% A; 65 min, 60% B∶40% C; 36 min, 100% A; 65 min, 60% B∶40% B; 36 min, 100% A; 65 min, 60% B∶40% B; 100% A; 65 min, 60% B∶40% C; 100 min, 60% B∶40% C. The flow rate was 1 mL·min^−1^, the column temperature was 30 °C, the detection wavelength was 248 nm, and the injection volume was 10 μL. The contents of arginine in the fruits were calculated from the standard curves using the arginine standard under the same chromatographic conditions in the unit of μmol·g^−1^.

The polyamine content was determined by the method of Yamaguchi et al. [38], with slight modification. About 0.5 mycelium was weighed, and 4 mL of pre-cooled 5% (*v*/*v*) perchloric acid (PCA) was added to the homogenate on ice. After an ice bath for 1 h and centrifugation (FRESCO21, Thermo Fisher Scientific, Sunnyvale, CA, USA) at 15,000× *g* for 30 min at 4 °C, 500 μL of the supernatant was taken, and 1 mL of NaOH (2 mol·L^−1^) and 7 μL of benzoyl chloride were added and mixed, and then the supernatant was vigorously vortexed and incubated in a warm bath at 37 °C for 30 min. The reaction was terminated by adding 2 mL of saturated NaCl solution to the mixture, then 2 mL of ether was added for extraction, and centrifuged at 1500× *g* for 5 min. 1 mL of the ether phase was evaporated and dried with a rotary evaporator (Zhengzhou Biochemical Instrument Co., Ltd., Zhengzhou, China), and then dissolved in 400 μL of chromatographic-grade methanol to be measured. 40 μL of the three polyamines (Put, Spd, and Spm) were prepared into a storage solution of 1.0 mmol·L^−1^, and then benzoic acid chloride was used for the determination of polyamines in the same way as that of spermidine samples. The polyamines were determined by high performance liquid chromatography (LC-2010AHT, Japan; C18 reversed-phase column, 4.6 mm in diameter, 150 mm in length, and 5 μm in particle size) with an injection of 10 μL, a column temperature of 25 °C, a flow rate of 0.7 mL·min^−1^, and the detection of the absorption peaks under the UV light of 230 nm with 64% methanol as the mobile phase. The peak absorption was detected at 230 nm under the ultraviolet light. For the standard curve, the benzoylated polyamine standard solution was injected into the sample at 0.1, 0.2, 0.3, 0.4 nmol·μL^−1^, and the correlation coefficients were calculated as the peak area versus the injection volume.

#### 2.6.3. Detection of Put on Activities of ADC, ODC, DAO, and PAO

ADC and ODC activities were determined with reference to the method of Zhang et al. [39]. 2 g of mycelium was homogenized by adding 5 mL of 100 mmol·L^−1^ pH 8.0 sodium phosphate buffer containing 0.1 mmol·L^−1^ phenylmethyl sulfonylfluoride (PMSF), 1 mmol·L^−1^ pyridoxal phosphate (PLP), 5 mmol-L^−1^ dithiothreitol (DTT), 5 mmol·L^−1^ ethylene diamine tetraacetic acid (EDTA), 25 mmol·L^−1^ ascorbic acid and 1% polyvinyl polypyrrolidone (PVPP), and the supernatant was taken as the crude enzyme extract. After homogenization, the supernatant was centrifuged (FRESCO21, Thermo Fisher Scientific, Sunnyvale, CA, USA) at 4 °C and 12,000× *g* for 20 min, and the supernatant was used as the crude enzyme extract. The reaction system was constructed by taking 1.5 mL of 10 mmol·L^−1^ Tris-HCl buffer (containing 5 mmol·L^−1^ EDTA, 50 μmol·L^−1^ PLP and 5 mmol·L^−1^ DTT) and 0.3 mL of crude enzyme extract, and 0.2 mL of 25 mmol·L^−1^ arginine (or ornithine) was added at the beginning of the reaction for the determination of ADC activity (or OC activity). The reaction was started with 0.2 mL of 25 mmol·L^−1^ arginine (or ornithine) for the determination of ADC activity (or ODC activity). After mixing, the reaction was terminated by adding phosphoric acid solution after 1 h at 37 °C. Finally, the reaction mixture was centrifuged at 5000× *g* for 10 min, and the supernatant was measured at 254 nm to determine the absorbance value in the microplate reader (BoTeng, BioTek, Winooski, VT, USA). 0.01 change in absorbance per minute per gram of mycelium was taken as one unit of enzyme activity (U) for ADC and ODC activities, which was expressed as U·g^−1^.

DAO and PAO activities were determined by the method of Palma et al. [40]. A total of 2 g of mycelium was homogenized by adding 5 mL of 100 mmol phosphate buffer pH 6.5, and then centrifuged at 12,000 × *g* for 20 min at 4 °C, and the supernatant was used as the crude enzyme extract. The reaction system was constructed with 2.0 mL of 100 mmol·L^−1^ pH 6.5 phosphate buffer, 0.2 mL of color reagent (containing 25 μL of N, N-dimethylphenylamine and 10 mg·100 mL^−1^ of 4-aminoantipyrine), 0.1 mL of 250 U·mL^−1^ horseradish peroxidase, and 0.5 mL of the crude enzyme extract. 0.2 mL of 20 mmol·L^−1^ Put (or Spd and Spm) was added for the determination of DAO (or PAO) activity, and the change in absorbance was measured at 550 nm after homogenization in a microplate reader (BoTeng, BioTek, Winooski, VT, USA). The activity of DAO and PAO was expressed in U·g^−1^ as a unit of enzyme activity (U) with a change in absorbance of 0.01 per gram of mycelium per minute.

#### 2.6.4. Measurement of Endogenous NO, and NOS Activity

Endogenous NO was determined using the NO assay kit (S0021S, Beyotime Biotechnology, Shanghai, China) according to the instructions. The measured NO content of the kiwifruit samples was expressed as μmol g^−1^ protein. The NOS activity was determined using a nitric oxide synthase (NOS) type assay kit (S0025, Beyotime Biotechnology, Shanghai, China) according to the manufacturer’s instructions. The measured NOS activity of the kiwifruit samples was expressed as U mg^−1^ protein.

#### 2.6.5. Detection and Characterization of Endogenous NO and ROS

**Figure 1:**
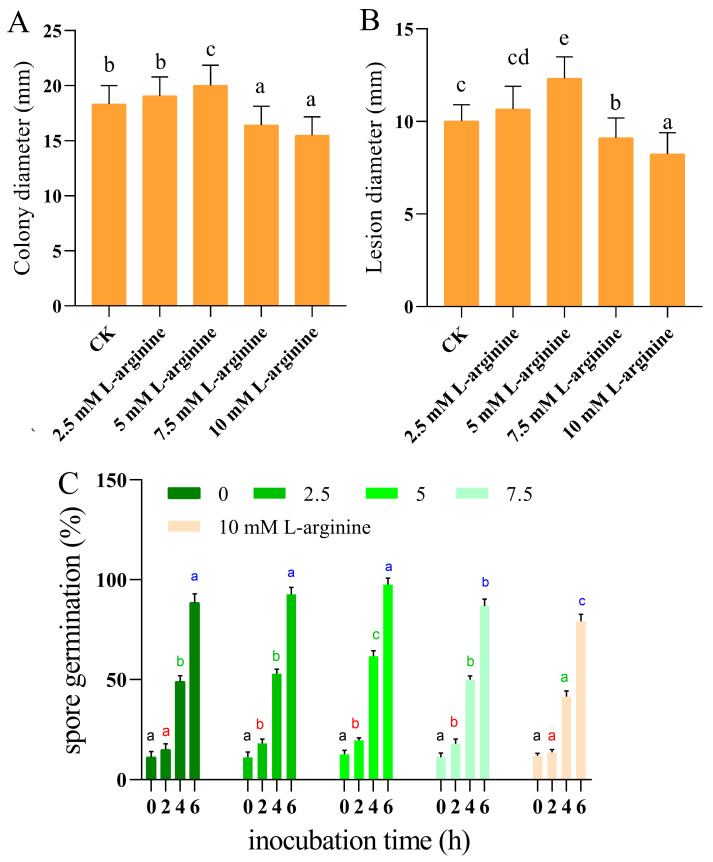
Effect of L-arginine on colony diameter in vitro (**A**), diameter of black spot disease in vivo (**B**), and spore germination rate of kiwifruit (**C**). Values are presented as means ± SD (*n* = 10). Different letters indicate significant differences (*p* < 0.05).

**Figure 2:**
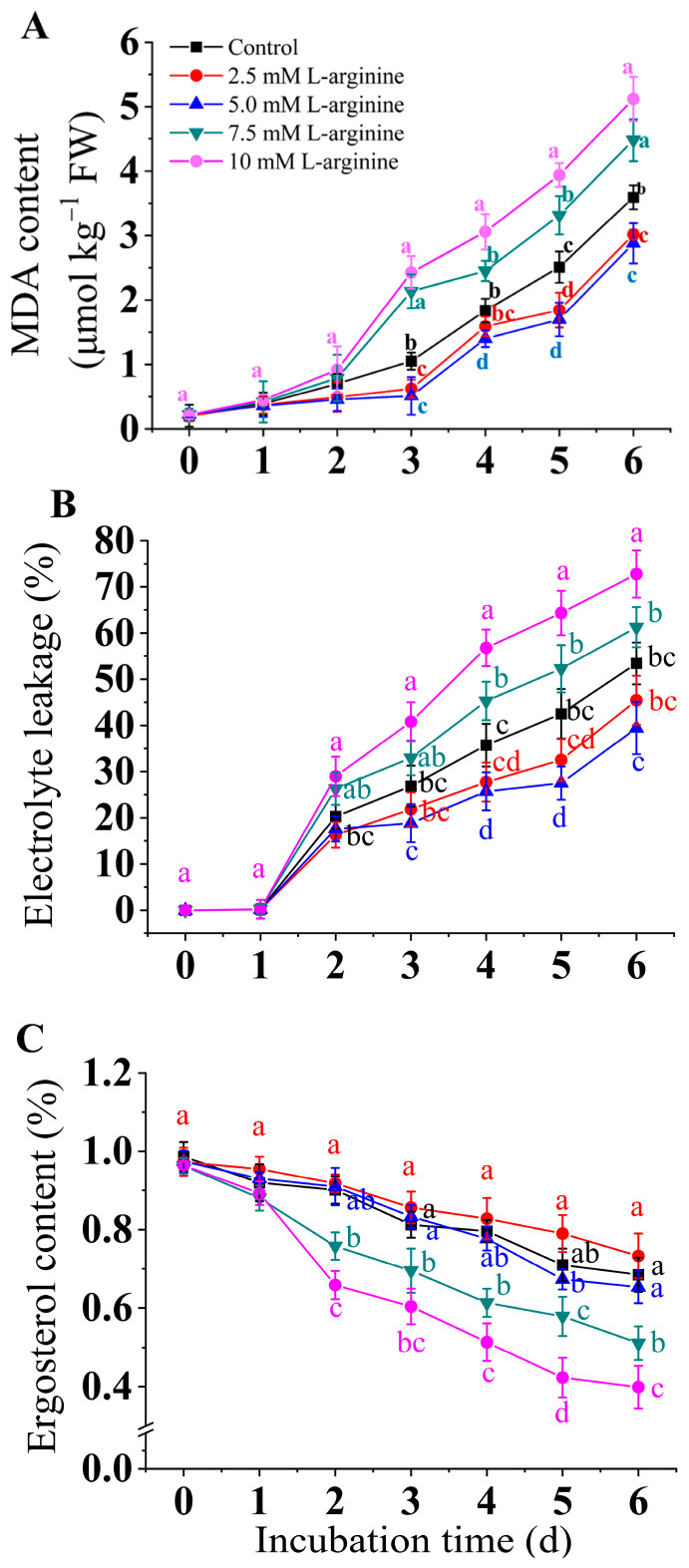
Effect of L-arginine on MDA content (**A**), electrolyte leakage (**B**), and ergosterol content (**C**) of *A. alternata*. Values are presented as means ± SD (*n* = 10). Different letters indicate significant differences (*p* < 0.05).

According to the method of Hu et al. [41]. The NO and ROS were detected by 3-amino, 4-aminomethyl-2′, 7′-difluorescein diacetate (DAF-FM) and 2, 7-dichlorodi -hydrofluorescein diacetate (DCHF-DA) fluorescence staining, respectively. Based on the previous study (Figure 1 and Figure 2), the spore suspension of *A. alternata* was added to PDB medium containing 5.0 mM L-arginine, and distilled water treatment was used as a control. The spores were collected by centrifugation (FRESCO21, Thermo Fisher Scientific, Sunnyvale, CA, USA) at 12,000× *g* for 5 min after incubation at room temperature for 3 h in biochemical incubator (MJP 250, Shanghai Keheng Industrial Development Co., Ltd., Shanghai, China).

The spores were then rinsed twice with phosphate-buffered saline (PBS) buffer (pH 7.4), and the fluorescent probes DAF-FM and DCHF-DA were added and adjusted to concentrations of 5μM L^−1^ and 20 μg L^−1^, respectively. Dark incubation at 37 °C for 20 min and 30 °C for 60 min was used for NO and ROS detection, respectively. The spores were then rinsed twice with PBS buffer, and then observed and photographed under the fluorescence microscope (DM 2500, Leica, Heidelberg, Germany). Three replicates were used for each group.

#### 2.6.6. The Rate of O_2_^−^ Production and H_2_O_2_ Content Assay

A H_2_O_2_ content assay was performed using a hydrogen peroxide (H_2_O_2_) assay kit (S0038-1, Beyotime Biotechnology, Shanghai, China) according to the manufacturer’s instructions. The measured H_2_O_2_ content of the samples was expressed as mmol g^−1^ protein.

The generation rate of O_2_^.−^ was assayed using a super anion activity content assay kit (BC1290, Solarbio Science and Technology, Beijing, China) according to the manufacturer’s instructions. The measured generation rate of the O_2_^.−^ of the samples was expressed as min^−1^ g^−1^ FW.

#### 2.6.7. Detection of NOX, SOD, CAT, POD, APX, and GR Activities

A NOX activity assay was performed using a NADHP oxidase (NOX) assay kit (S0086, Beyotime Biotechnology, Shanghai, China) according to the manufacturer’s instructions. The measured NOX activity of the samples was expressed as U mg^−1^ protein.

A SOD activity assay was performed using a superoxide dismutase (SOD) assay kit (S0086, Beyotime Biotechnology, Shanghai, China) according to the manufacturer’s instructions. The measured SOD activity of the samples was expressed as U mg^−1^ protein.

CAT activity was performed using a catalase (CAT) assay kit (P3541, Beyotime Biotechnology, Shanghai, China) according to the manufacturer’s instructions. The measured CAT activity of the samples was expressed as U mg^−1^ protein.

POD activity was performed using a peroxidase assay kit (076323, Shanghai Enzyme-linked Biotechnology Co., Ltd., Shanghai, China) according to the manufacturer’s instructions. The measured POD activity of the samples was expressed as U mg^−1^ protein.

GPX activity was performed using a glutathione peroxidase (GPX) activity assay Kit (S0038-1, Beyotime Biotechnology, Shanghai, Chinaa) according to the manufacturer’s instructions. The measured GPX activity of the samples was expressed as U mg^−1^ protein.

GR activity was performed using a glutathione reductase assay kit (092942, Shanghai Enzyme-linked Biotechnology Co., Ltd., Shanghai, China) according to the manufacturer’s instructions. The measured GR activity of the samples was expressed as U mg^−1^ protein.

### 2.7. Detection of Cx, β-1,3-Glucanase, PG, PMG, PGTE, and PMTE Activities In Vitro and In Vivo

#### 2.7.1. Extraction and Purification of Crude Enzyme Solution In Vitro

The assessment of the CWDE activities was based on the method of Ge et al. [33]. with minor modifications. *A. alternata* was incubated on a PDA medium for 7 d in a biochemical incubator (MJP 250, Shanghai Keheng Industrial Development Co., Ltd., Shanghai, China) at 28 °C in the dark, and then agar disks (6 mm diameter) containing *A. alternata* mycelium were taken with a punch. The agar disks containing *A. alternata* mycelium were inoculated into 250 mL of PDB medium (containing 0 mM and 5.0 mM L-arginine, respectively) and incubated at 25 °C at 150 rpm min^−1^ with constant temperature. The mycelium was filtered, and the medium solution was collected on days 0, 1, 2, 3, 4, and 5, respectively. The medium solution was centrifuged (FRESCO21, Thermo Fisher Scientific, Sunnyvale, CA, USA) at 12,000× *g* for 30 min at 4 °C, and the supernatant was extracted to determine the enzyme activity.

The crude enzyme solution was mixed with 60% saturated ammonium sulfate and let to stand at 4 °C for 5 h. After centrifugation at 15,000× *g* for 25 min at 4 °C, the precipitate was collected and dissolved in 50 mM of acetic acid–sodium acetate buffer (pH 5.0), and then dialyzed at 4 °C for 48 h to obtain the purified enzyme solution.

#### 2.7.2. Extraction and Purification of Crude Enzyme Solution In Vivo

The *A. alternata* was cultured on potato dextrose agar (PDA) for seven days, and the spore suspension (10^6^ spores × 10^−3^ L^−1^) was prepared for inoculation. A total of 300 kiwifruits were selected and randomly divided into two groups of 150 (L-arginine-treated group and control group), 3 replicates of 50 each. The fruit surface was sterilized with 75% alcohol, then spread out on the test bench to allow the surface moisture to dry naturally, and then uniformly punched on the surface of the tubers with a perforator (4 mm in diameter). The holes were inoculated with 20 μL of spore suspension, dried, and stored at 22 (±2 °C). Tissue samples of the *A. alternata* were collected from the onset site areas of the fruit on days 0, 1, 2, 3, 4, and 5 after inoculation, respectively.

The samples (1 g) were added to 9 mL of 1 mol L^−1^ NaCl and homogenized at 0 °C. The homogenate was centrifuged at 4 °C and 12,000× *g* for 20 min, and the supernatant was collected and stored at 4 °C. The kiwifruits were inoculated with spore suspensions treated with sterile water in PDB medium as a control. The difference between the activities of the CWDEs in the *A. alternata* inoculated with L-arginine-treated spores and those inoculated with sterile water-treated spores was used to represent the activity of the CWDEs secreted by *A. alternata* during infection.

#### 2.7.3. Measurement of CWDEs’ Activities

Cx activity was performed using a cellulase assay kit (095198, Shanghai Enzyme-linked Biotechnology Co., Ltd., Shanghai, China) following the manufacturer’s instructions. The measured Cx activity of the samples was expressed as U mg^−1^ protein.

β-1,3-glucanase activity was performed using a β-1,3-glucanase assay kit (MC574L, Shanghai Enzyme-linked Biotechnology Co., Ltd., Shanghai, China) following the manufacturer’s instructions. The measured β-1,3-glucanase activity of the samples was expressed as U mg^−1^ protein.

PG activity was performed using a polygalacturonase assay kit (076398, Shanghai Enzyme-linked Biotechnology Co., Ltd., Shanghai, China) following the manufacturer’s instructions. The measured PG activity of the samples was expressed as U mg^−1^ protein.

The PMG activity reaction system consisted of 0.5 mL of 1.0 mg mL^−1^ pectin, 1.0 mL of 50 mM L^−1^ acetate buffer (pH 5.5), and 0.5 mL of crude enzyme solution. The reaction solution was incubated in a water bath (XMTD-4000, Shanghai Keheng Industrial Development Co., LTD., Shanghai, China) at 37 °C for 1 h. After cooling, 1.0 mL of 3,5-dinitrosalicylic acid (DNS) was added rapidly, boiled for 5 min, and then rapidly cooled to room temperature. Sterile water was used instead of the crude enzyme solution as a control, and the absorbance value at 540 nm was measured after cooling to room temperature in a microplate reader (BoTeng, BioTek, Winooski, VT, USA). The PMG activity was expressed as U mg^−1^ protein.

The PMTE and PGTE reaction system consisted of 1.0 mL 3.0 mmol L^−1^ CaCl_2_, 4.0 mL 50 mmol L^−1^ glycine, sodium hydroxide buffer (pH 9.0), 3.0 mL 1.0 g L^−1^ reaction substrate (with the substrates of PMTE and PGTE being pectin and polygalacturonic acid, respectively), and 0.1 mL of crude enzyme solution. The reaction system solution was incubated in a water bath (XMTD-4000, Shanghai Keheng Industrial Development Co., Ltd., Shanghai, China) at 30 °C for 10 min and then cooled down, and the absorbance value at 232 nm was determined after cooling to room temperature in a microplate reader (BoTeng, BioTek, Winooski, VT, USA). The crude enzyme solution was replaced with sterile water as a control. The activities of PGTE and PMTE were expressed as U mg^−1^ protein.

The total protein content was measured by using Coomasse Brilliant Blue staining [42].

### 2.8. Gene Expression Analysis by Quantitative Real-Time PCR (RT-qPCR)

The total RNA was isolated from ground tissues and first-strand cDNA was synthesized by the cetyltrimethylammonium bromide (CTAB) method and extracted using a Takara RNA extraction kit (Takara Biotechnology, Japan). The RT-qPCR was performed for the expression levels of *A. alternata NOS* (*AaNOS)*, *AasGC*, *AaNOXa*, *AaNOXb*, *AaSOD*, *AaCAT*, *AaAPX*, *AaGR*, *AaCx*, *Aa*β-1,3-glucanase, and *Aa*PG, with primer information for the amplification of the above genes given in Table 1. The SYBR Green PCR Premix Ex Taq™ (Takara Biomedicals, Shiga, Japan), cDNA, forward and reverse primers, and ROX reference dye II, were added to an ABI 7000 instrument (Applied Biosystems, Foster City, California, USA) for reaction. The operation was as follows: at 95 °C for 10 s, at 95 °C for 5 s with 40 cycles, and at 60 °C for 40 s. The *Alternaria alternata actin (Act1) gene* (*PbActin*, MN164690.1) was used as an internal reference. The relative quantifications were then calculated using the 2^−△△CT^ method and the CT values from the *S. tuberosum* actin gene were used to normalize all the RT-qPCR reactions.

### 2.9. Data Analysis

Each treatment included three biological replicates, and the data were analyzed using the SPSS11.0 software package (SPSS Inc., Chicago, IL, USA). The data were subjected to a one-way analysis of variance (ANOVA) and Duncan’s post hoc test, with the significance set at a *p*-value < 0.05. The results were presented as the mean ± standard deviation. Heat maps were used to visualize the expression level of each gene using GraphPad Prism8.0 software (GraphPad Software, San Diego, CA, USA).

## 3. Results

### 3.1. Effect of the L-Arginine on the Growth of Mycelium In Vitro and In Vivo and on the Spore Germination of A. alternata

As shown in Figure 1A, the low concentration (2.5 mM) of L-arginine treatment did not significantly inhibit or promote the growth of *A. alternata* from the pre-growth stage until the late growth stage in vitro, and there was no significant change in the colony diameter of the L-arginine-treated group compared to the control group (*p* < 0.05). When the concentration of L-arginine was increased to 7.5 mM, the colony diameter was significantly reduced, and the growth of *A. alternata* was inhibited (*p* < 0.05), and the growth of *A. alternata* in the 7.5 mM and 10 mM arginine-treated groups was inhibited by 8.8% and 17.6% compared to the control group (Figure 1A). The results of the in vivo experiment showed that when the concentration of L-arginine was increased to 10 mM L^−1^, it showed significant inhibition of kiwifruit black spot disease on the damaged inoculated kiwifruits, and the diameter of the kiwifruit spots in the L-arginine-treated group was reduced by 15.55% compared to the control (Figure 1B), and below this concentration, there was no obvious effect.

The results of spore germination showed that the spore germination rate was significantly higher than that of the control group at low concentrations of L-arginine (*p* < 0.05), and the spore germination rate of the 5.0 mM L-arginine-treated group was 25.9% higher than that of the control group at the fourth hour. When the L-arginine was increased to 10 mM, the *A. alternata* spore germination was inhibited, showing a slightly delayed effect (Figure 1C).

### 3.2. Effect of L-Arginine on Electrolyte Leakage, MDA and Ergosterol Contents

As is shown in Figure 2A, the MDA contents increased in all the groups at the early stage. The MDA content in the 10 mM and 7.5 mM L-arginine treated groups was higher than the control group during the end of the inoculation period, which was 42.6% and 24.8% higher than the control, respectively. In the 5.0 mM L-arginine treated group, the increase in MDA content slowed down. The changing trend of electrolyte leakage content was similar to that of the MDA. At the end of the incubation period, the electrolyte leakage of the 5.0 mM L-arginine-treated group was 35.5% lower than that of the control (Figure 2B). In all the groups, the ergosterol contents gradually decreased with the incubation time. Figure 2C shows that 5.0 of mM L-arginine treatment significantly prevented the decrease in the ergosterol content (*p* < 0.05). After 5 days, the 7.5 mM and 10 mM L-arginine-treated groups were 33.3% and 74.4% lower than the control group, while the 2.5 and 5.0 mM-treated groups had no significant difference from the control group.

### 3.3. Effect of L-Arginine on Contents of Endogenous Arginine, Put, Spd, and Spm

**Figure 3:**
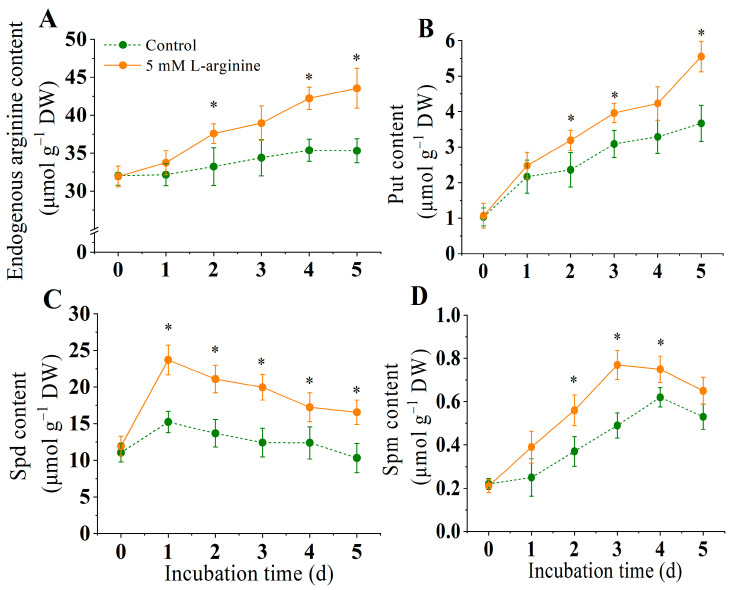
Effect of L-arginine on arginine content (**A**), Put content (**B**), Spd content (**C**), and Spm content (**D**) of *A. alternata*. Values are presented as means ± SD (*n* = 10). * indicates significant differences (*p* < 0.05).

As shown in Figure 3A, the exogenous addition of 5.0 mM L-arginine made the arginine content in *A. alternata* continue to rise during the treatment period, significantly higher than that of the control group (*p* < 0.05). On day 5, the endogenous arginine content of the treatment group was 2.3 times that of the control group. The exogenous addition of 5.0 mM L-arginine significantly promoted the accumulation of Put, Spd, and Spm (*p* < 0.05) (Figure 3B–D). The Put content continued to rise throughout the treatment period, and the Put content was significantly higher than the control group and 1.5 times higher than that of the control group at the end of the incubation time (Figure 3B). The Spd content of both the arginine-treated group and the control group increased rapidly and peaked after one day of culture. At this time, the Spd content of the treated group was 1.6 times that of the control group, but the arginine-treated group was always significantly higher than that of the control group (Figure 3C). The Spm content increased first and then decreased during the culture period. The Spm content of the control group peaked in the fourth day, and the arginine-treated group peaked on day 3, when the Spm content of the treated group was 1.2 times that of the control group (Figure 3D).

### 3.4. Effect of L-Arginine on Activities of ADC, ODC, DAO, and PAO

**Figure 4:**
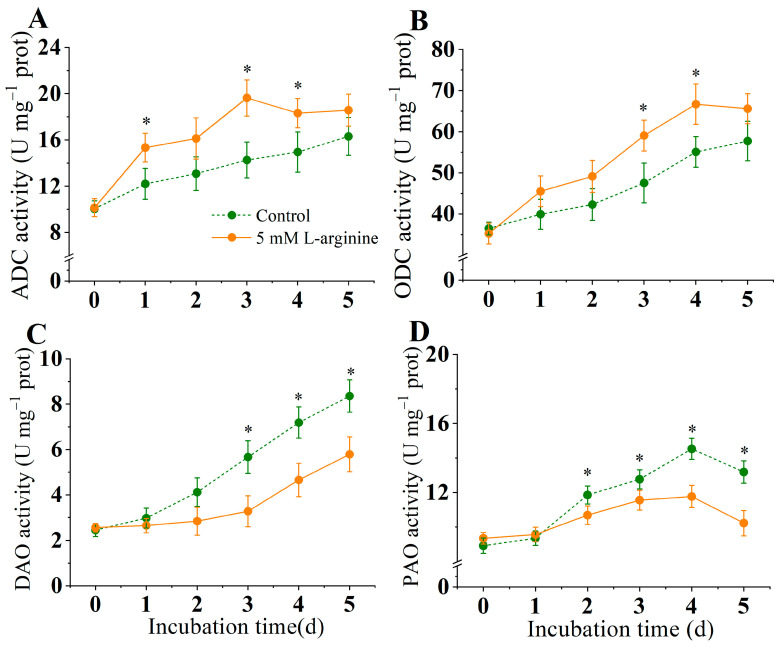
Effect of L-arginine on activities of ADC (**A**), ODC (**B**), DAO (**C**), and PAO (**D**) of *A. alternata*. Values are presented as means ± SD (*n* = 10). * indicates significant differences (*p* < 0.05).

Figure 4 shows that 5.0 mM L-arginine significantly activated the ADC and ODC activities and reduced the DAO and PAO activities compared to the control group (*p* < 0.05). The ADC and ODC activities gradually increased before the arginine phase and peaked on days 3 and 4, 37.6% and 20% higher than the control group, respectively, while the control ADC and ODC activities increased throughout the incubation period but were consistently lower than the arginine-treated group (Figure 4A,B). Both the DAO and PAO activities showed a gradual increase in the early incubation period, but the enzyme activity of the arginine-treated group was always lower than that of the control group (Figure 4C,D). Arginine treatment reduced the DAO activity by 56.9% and the PAO activity by 26.8% on day 5, as compared to the control subjects.

### 3.5. Effect of L-Arginine on NOS Activity, and Endogenous NO Content

**Figure 5:**
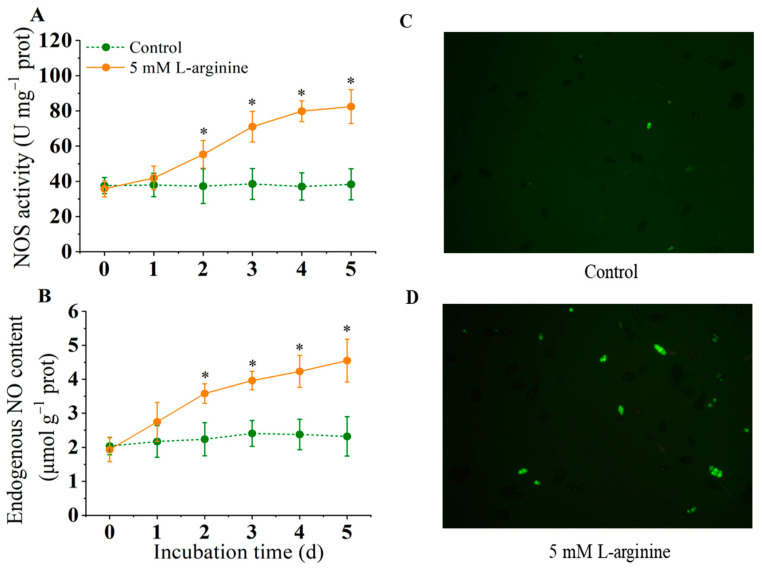
Effect of L-arginine on NOS activity (**A**), endogenous NO content (**B**), and NO fluorescence (**C**,**D**) of *A. alternata*. Values are presented as means ± SD (*n* = 10). * indicates significant differences (*p* < 0.05).

The results showed that 5.0 mM L-arginine activated the NOS activity significantly (*p* < 0.05) (Figure 5A); the NOS activity of the L-arginine-treated group had a rapid increase from day 2 of storage. Similarly, the endogenous NO content increased from day 2 to day 5 (Figure 5B). In the control group, there was almost no increase in the amplitude of NOS activity and endogenous NO accumulation. The *A. alternata* spores were stained with DAF-FM fluorescent probe, and NO accumulation was observed via confocal microscopy (*p* < 0.05) (Figure 5D). The fluorescence staining results showed that 5.0 mM L-arginine treatment significantly promoted the production of endogenous NO in the *A. alternata* spores, while the fluorescence intensity of the control group was significantly lower than that of the L-arginine-treated group, which was consistent with the changes in the NO content *in* the *A. alternata*.

### 3.6. Effect of L-Arginine on the Generation of O_2_^−^ and H_2_O_2_ Content

**Figure 6:**
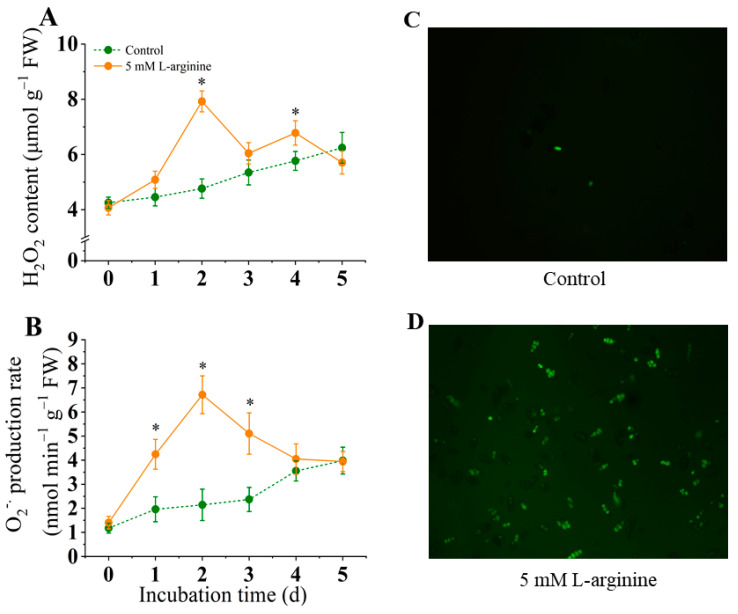
Effect of L-arginine on endogenous H_2_O_2_ content (**A**), production rate of O_2_^−^ (**B**), and ROS fluorescence (**C**,**D**) of *A. alternata*. Values are presented as means ± SD (*n* = 10). * indicates significant differences (*p* < 0.05).

As shown in Figure 6A, the H_2_O_2_ content in the L-arginine-treated group initially increased rapidly from day 0 to day 2, when the H_2_O_2_ content of the 5.0 mM L-arginine-treated group was 15.9% higher than that of the control group, and then gradually decreased. In the control group, the H_2_O_2_ content gradually increased from 0 to day 5. In Figure 6B, the O_2_^.−^ production rate in the control group increased slightly from day 0 to day 5. In the L-arginine treatment group, it increased sharply from day 0 to day 2 and then decreased rapidly from day 3 to day 5. On day 2, the production rate of the O_2_^.−^. of the 5.0 mM L-arginine treatment was 2.2 times higher than that of the control group. On day 5, the O_2_^−^ production in the L-arginine treatment group was similar to that of the control group.

*A. alternata* spores were stained with the fluorescent probe DCFH-DA, and the accumulation of reactive oxygen species (ROS) was observed via confocal microscopy (Figure 6C,D). The fluorescence staining results showed that 5.0 mM L-arginine treatment significantly induced the accumulation of large amounts of ROS in the *A. alternata* spores, while the fluorescence intensity of the control group was significantly lower than that of the L-arginine-treated group, which was consistent with the changes in H_2_O_2_ and O_2_^−^.

### 3.7. Effect of L-Arginine on Activities of NOX, SOD, CAT, POD, GPX and GR

**Figure 7:**
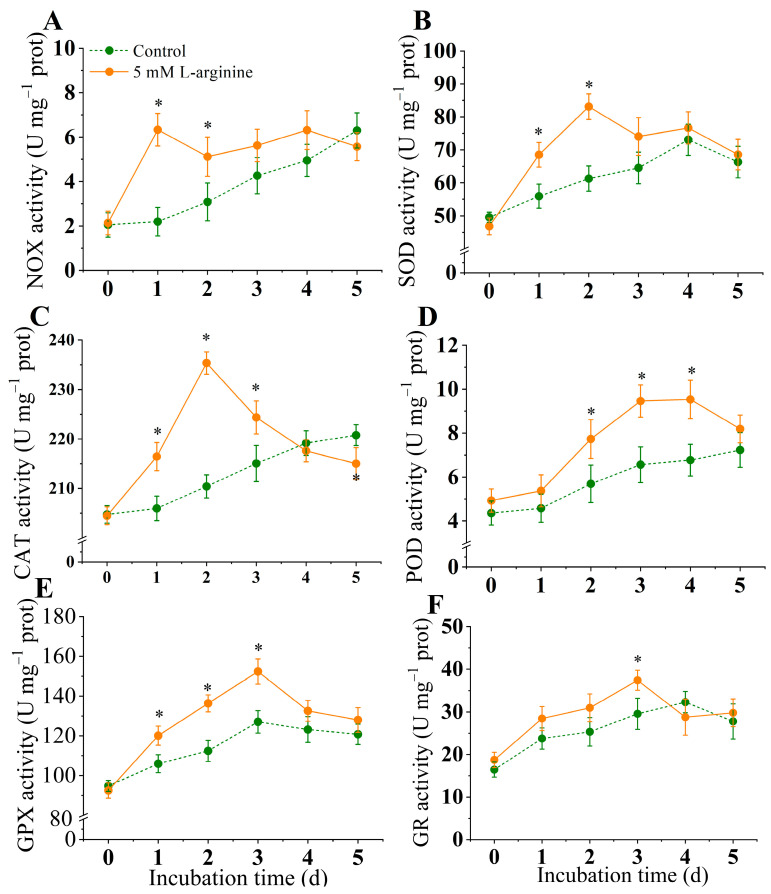
Effect of L-arginine on activities of NOX (**A**), SOD (**B**), CAT (**C**), POD (**D**), GPX (**E**), and GR (**F**) of *A. alternata*. Values are presented as means ± SD (*n* = 10). * indicates significant differences (*p* < 0.05).

The results showed that L-arginine could significantly activate the activity of NOX compared with the control group (*p* < 0.05) (Figure 7A). The NOX activity in the L-arginine-treated group peaked on day 1 and then decreased. On day 1, the NOX activity was 2.9 times higher than that of the control group.

As shown in Figure 7B, the SOD activity in the L-arginine-treated group increased and then decreased during the incubation period and was markedly higher than that in the control group during the 0–5 day period (*p* < 0.05). The SOD activity in the L-arginine-treated kiwifruits peaked after day 2, whereas the SOD activity in the control group peaked on day 4. During the incubation time, the L-arginine treatment could effectively enhance the SOD activity in the *A. alternata*. The SOD activity of the L-arginine-treated group was higher than that of the control group, and the greatest difference in SOD activity was observed at day 2, when the L-arginine -treated group was 22.5% higher than the control group.

The CAT activity in the L-arginine-treated group was significantly higher than that in the control group from day 1 to day 3, and lower than that in the control group at the end of the incubation time (*p* < 0.05) (Figure 7C). The results showed that the L-arginine enhanced POD activity of mycelium, which increased steadily from day 0 to day 3, where it reached the peak value, and began to decline on day 4. In the control group, the POD activity continued to increase from day 0 to day 5; however, the overall level of POD activity was lower than those in the L-arginine-treated group (Figure 7D).

The GPX activity in the L-arginine-treated group increased from day 0 to day 3; the highest peak of GPX activity in both treatments on day 3 was 127.11  ±  3.2 U for the control and 152.36  ±  4.8 U for the L-arginine treatment, respectively (Figure 7E). As shown in Figure 7F, the GR activity of the control group was generally more stable than that of the treatment group. After treatment with L-arginine, the GR activity of the mycelium increased significantly (*p* < 0.05), reaching a peak on day 3, which was 26.6% higher than that of the control group. Subsequently, the GR activity decreased, and the GR activity of the L-arginine-treated group was lower than that of the control group on day 5.

### 3.8. Effect of L-Arginine on the Activities of CWDEs from A. alternata

#### 3.8.1. CWDEs from *A. alternata* In Vitro

**Figure 8:**
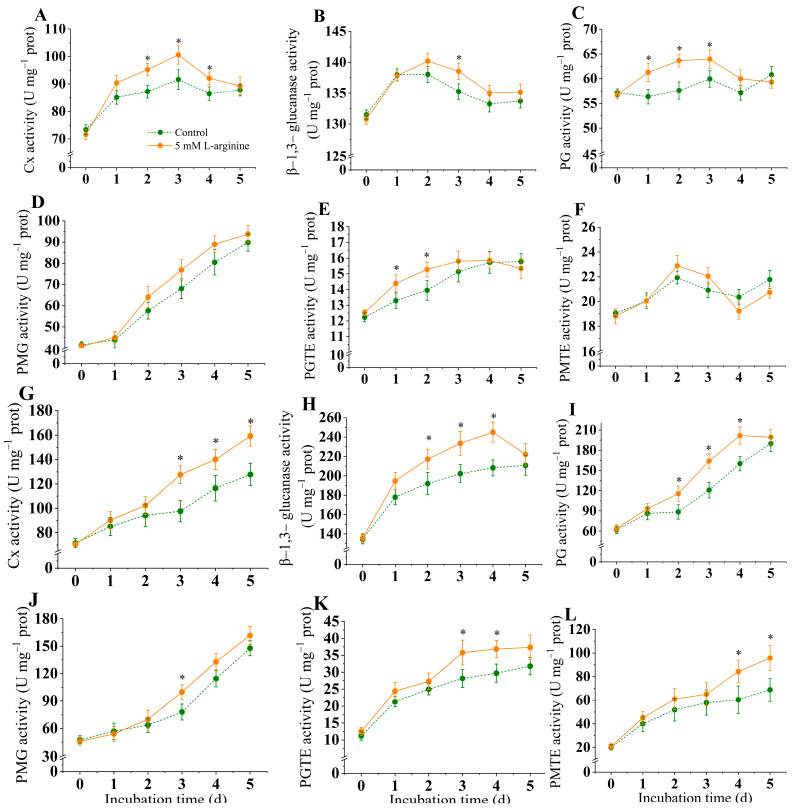
Effect of L-arginine on activities of Cx (**A**), β-1,3-glucanase (**B**), PG (**C**), PMG (**D**), PGTE (**E**), and PMTE (**F**) from *A. alternata* in vitro, and Cx (**G**), β-1,3-glucanase (**H**), PG (**I**), PMG (**J**), PGTE (**K**), and PMTE (**L**) from *A. alternata* of kiwifruit, respectively. Values are presented as means ± SD (*n* = 10). * indicates significant differences (*p* < 0.05).

Figure 8 showed the changes in the cell wall-degrading enzymes of *A. alternata* in vitro. Compared to the control group, L-arginine treatment significantly induced a significant increase in the Cx and β-1,3-glucanase activities (*p* < 0.05). Cx and β-1,3-glucanase activities increased in the early inoculation period and reached a peak on day 3 and day 2, respectively, at which time, the Cx and β-1,3-glucanase activities of the L-arginine-treated group were 9.8% and 1.6% higher than those of the control group, after which the Cx and β-1,3-glucanase activities gradually decreased (Figure 5A,B).

The PG and PGTE activities increased on days 0–3 and peaked on day 3, at which time the PG in the L-arginine-treated group was 8.5% higher than that in the control group, after which the PG and PGTE activities gradually decreased (Figure 8C,E). The PMG activities gradually increased with time and continued to increase during the incubation period (Figure 5D). Meanwhile, there was no significant difference in the PMG enzyme activity compared to the control (*p* < 0.05).

#### 3.8.2. CWDEs from *A. alternata* of Kiwifruit

Figure 8 shows the changes in the cell wall-degrading enzymes of the *A. alternata* inoculated in kiwifruits. Compared to the control group, L-arginine treatment significantly induced an increase in the Cx and β-1,3-glucanase activities; the Cx activities gradually increased with time and continued to increase during the incubation period, with 25.2% higher Cx activity than the control on day 5. The β-1,3-glucanase activities increased in the early inoculation period and reached a peak on day 4, at which time the β-1,3-glucanase activities of the L-arginine-treated group were 17.8% higher than those of the control group (Figure 8G,H).

The PG, PMG, PGTE, and PMTE activities gradually increased with time and continued to increase during the incubation period (Figure 8I–L). Compared with the control group, the increase in PG activity was significantly induced by L-arginine (*p* < 0.05), and the PG activity of the L-arginine-treated group was 35.8%, and higher than those of the control group on day 3 after inoculation, respectively (Figure 8I). The PMTE activity of the L-arginine treatment group was significantly higher than that of the control group at the later stage of inoculation in vivo (*p* < 0.05) (Figure 8L).

### 3.9. Effect of L-Arginine on Relative Gene Expression Levels of A. alternata

**Figure 9:**
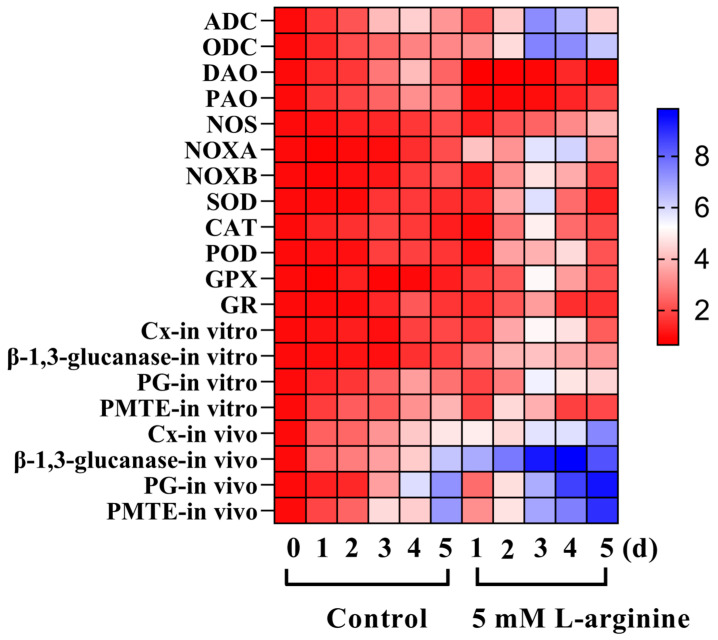
Effect of L-arginine on relative gene expression levels of *A. alternata.* Values are presented as means ± SD (*n* = 10).

RT-qPCR results showed that 5.0 mM L-arginine induced a significant up-regulation in the expression of *AaADC*, *AaODC*, *AaNOXA*, *AaNOXB*, *AaSOD*, *AaCAT*, *AaPOD*, and *AaGPX*, and no significant change in the expression of *AaDOA* and *AaPOA* compared to the control group (*p* < 0.05) (Figure 9). On day 3, the expression levels of *AaADC* and *AaODC* were 1.84 times and 3.01 times higher in the L-arginine-treated group than those in the control group, respectively. Meanwhile, the level of *AaNOXA* gene expression in the L-arginine-treated group was 3.68 times higher than in the control group. The results of the in vitro experiments showed that 5.0 mM L-arginine significantly induced the up-regulation of *AaCx*, *Aaβ-1,3-glucanase*, *AaPMG*, and *AaPMTE* expression, and the change in AaPG expression was not significant. The results of he vivo experiments showed that the *AaCx*, *Aaβ-1,3-glucanase*, *AaPG*, and *AaPMTE* expression levels were significantly induced to be expressed by 5.0 mM L-arginine, and the expression of the CDWE-related genes after inoculation in the kiwifruits was significantly higher than that of in vitro experiments (*p* < 0.05). On the fifth day of inoculation, the gene expression levels of *AaCx*, *Aaβ-1,3-glucanase*, *AaPG* and *AaPMTE* in the L-arginine-treated group were 56.2%, 31.6%, 29.7%, and 25.6% higher than those in the control group, respectively.

## 4. Discussion

*Alternaria alternata* is a common plant-pathogenic fungus that can cause the black spot disease of kiwifruit and seriously affect fruit quality, causing huge economic losses [2]. L-arginine is a functional amino acid involved in the growth and development, spore formation, and host infection of plant-pathogenic fungi. It has been reported that arginine can induce mycelium formation and dimorphic transformation in *Ceratocystis ulmi*, which causes Dutchelm disease [43]. In addition, pathogenic fungi with impaired arginine synthesis, such as *M. oryzae*, *F. oxysporum* showed a decrease in growth, development, and pathogenicity [3,4]. In this study, we confirmed that 5.0 mM of L-arginine could effectively promote mycelial growth and spore germination in vitro of *A. alternata* (Figure 1). An inoculation experiment in kiwifruit showed that 2.5 mM of L-arginine had no obvious inhibition or promotion effect on mycelia growth in *A. alternata*. When the concentration of arginine increased to 7.5 mM, the growth of *A. alternata* was inhibited (Figure 1B). Previous studies have confirmed high concentrations of L-arginine-inhibited growth on *B. cinerea*, while low concentrations of L-arginine promoted the mycelial growth of *Hypsizygus marmoreus* and *Colletotrichum coccodes* [44,45]. Meanwhile, as a substrate for the synthesis of NO, the important function of low levels of L-arginine in regulating spore germination of fungi has also been proven. Research reported that low levels of L-arginine facilitated the growth and development and spore germination of *B. cinerea* [44]. In contrast, excessive L-arginine inhibited spore germination in *B. cinerea* [44]. Our study showed similar results. The present study showed that 2.5 mM and 5 mM L-arginine significantly increased spore germination in *A. alternata* (*p* < 0.05). However, 10 mM of L-arginine had the opposite effect, delaying germination (Figure 1C). Therefore, we speculate that a low concentration of L-arginine promotes the mycelial growth and spore germination of *A. alternata* by regulating a series of signal transductions related to NO in fungal cells, while excessive L-arginine will produce cytotoxicity, affecting the cellular activities of fungi and inhibiting their growth and development [44]. Also, changes in the intracellular NO levels of *A. alternata* indirectly supported this conclusion (Figure 5B).

Ergosterol, an isoprenoid derivative, is the major sterol component of fungal cell membranes and is generally present in the free state in the phospholipid bilayer, helping to maintain the stability, integrity, and fluidity of the fungal cell membrane structure. It has been demonstrated that ergosterol reduction causes changes in sterol fractions, which disrupt the cellular structure and affect the operation of the normal function of the plasma membrane of fungal cells [46,47]. Figure 2 shows that treatment with 2.5 mM L-arginine significantly slowed down the decrease in ergosterol content in *A. alternata*, whereas treatment with high concentrations of L-arginine (7.5 mM and 10 mM) inhibited its synthesis. MDA content reflects the lipid peroxidation of cell membranes, and electrolyte leakage is one of the most important indicators of cell membrane integrity [41,46]. Our results indicated that treatment with a low concentration of L-arginine (2.5 mM and 5 mM) treatments reduced MDA content and electrolyte leakage in *A. alternata* and slowed the decline of ergosterol content, suggesting that low concentrations of arginine treatment help maintain the stability of the *A. alternata* cell membrane. However, MDA content and the electrolyte leakage of *A. alternata* were higher in the high-concentration L-arginine-treated group. Similar results were reported in our previous study on *B. cinerea* [44]. In *Penicillium digitatum* [47] and *Penicillium expansum* [48], ergosterol depletion was found to cause damage to the cell structure, especially cell membrane integrity, which was accompanied by increased MDA content and electrolyte leakage, which confirmed our results.

Polyamines are important polycations involved in the entire process of fungal growth and development. In this study, the addition of exogenous L-arginine (5.0 mM) significantly increased the arginine content in *A. alternata*, and the contents of putridine, arginine and, spermidine were also significantly increased due to the supplement of the precursor substance arginine (Figure 3). The intracellular levels of polyamines are strictly maintained through biosynthesis and degradation processes. Within fungi, polyamines can be synthesized directly through enzyme ornithine decarboxylase (ODC) or indirectly via arginine decarboxylase (ADC), while degradation occurs through diamine oxidase (DAO) and polyamine oxidase (PAO) [49]. Current research indicates that following a 5.0 mM L-arginine treatment, both the ODC and ADC activities were significantly enhanced, whereas the DAO and PAO activities were notably reduced (Figure 4). Consistent with this, the ADC and ODC genes were upregulated, while DAO and PAO were downregulated (Figure 9). This suggests that the supplementation of arginine promotes polyamine synthesis in *A. alternata* while inhibiting their degradation, leading to an accumulation of polyamines within the organism. It has been reported that polyamines affect spore germination and the hyphaehyphal branching of *Glomusetunicatum colonization* [12], the formation of appressorium in *M. oryzae* [11], and the production of mycotoxins in *Fusarium graminearum* [50]. In addition, polyamine synthesis was impaired in *Stagonospora nodorum* and *Ustilago maydis*, resulting in reduced pathogenic capacity [13,51]. Therefore, we speculated that the enhancement of the pathogenicity of L-arginine to Alternaria was dependent on the accumulation of polyamines in pathogenic fungi.

In the plant-pathogenic fungi, NO plays an important role in regulating cell growth, apoptosis and stress response. It has been reported that pathogenic fungi may use NO as a signaling molecule to infect plants. Samalova et al. [18] showed that the development of *M. oryzae* and the onset of infection were heavily dependent on the fungus NO synthesis. NO produced by *B. cinerea* can spread outside fungal cells, stimulating the colonization of pathogens in plant tissues [20]. NO is mainly produced by arginine catalyzed by NOS-like enzymes in the cytoplasm in fungi. [15] The use of NOS inhibitors in *C. coccodes*, *Phycomyces blakesleeanus*, etc., can reduce the level of NO in cells, confirming the existence of NOS enzymes in fungi and participating in the formation of NO [45,52]. The level of NO in *B. cinerea* cells increased with the increase in NOS activity [53]. Consistent with this, this study showed that 5.0 mM L-arginine significantly induced the increase in NOS activity and the expression of NOS genes, and increased endogenous NO levels. Given the positive role of NO in the growth and development of pathogenic fungi, we speculate that exogenous arginine may enhance the pathogenicity of *A. alternata* on kiwifruit by promoting the NOS pathway in endogenous arginine metabolism.

Fungal infection induces the massive production and accumulation of reactive oxygen species (ROS) in plants, which is also considered to be one of the earliest responses of host plants to pathogen invasion. Some studies have observed early ROS production in the host during the infection of various plants, such as *Arabidopsis thaliana* [54], tomato [55], and potato [41], which is considered to be one of the host resistance responses. However, it is interesting to note that ROS plays an important role in plant infection by pathogens [2]. *A. alternata* facilitates the infection process by forming infection structures and synthesizing ROS to destroy host plant tissues [2]. The host plant generates large amounts of ROS when attacked by pathogens to fight against the pathogen, and the pathogen responds to the oxidative stress by activating antioxidants [2]. *A. alternata* facilitate the infecting process via forming infection structures and synthesizing ROS to destroy host plant tissues [2]. The results of this study showed that the H_2_O_2_ content increased sharply on the second day after L-arginine treatment, peaked on the second and third day, respectively, and gradually decreased thereafter (Figure 6A). In addition, the O_2_^.−^ content in *A. alternata* peaked after two days of 5 mM L-arginine treatment and decreased thereafter (Figure 6B). H_2_O_2_ and O_2_^.−^ were observed to be rapidly induced at an early stage in this study, which may be one of the reasons for the proliferation of the pathogen (Figure 6) [2]. Moreover, in consideration of the coinstantaneous finding of an increase in SOD enzyme activity induced by L-arginine in *A. alternata* (Figure 7), the elevated H_2_O_2_ content in *A. alternata* can be attributed, on the one hand, to the inducing effect of L-arginine and, on the other hand, to the conversion of O_2_^.−^ to H_2_O_2_ by SOD [21].

NOX played an important role in the accumulation of ROS in pathogens by catalyzing oxygen molecules to O_2_^.−^, which contains both NoxA and NoxB family members. In fungi, genes encoding Nox-family enzymes have been found and associated with a wide range of functions in growth and development, physiological processes, and pathogenicity. In *Penicillium expansum*, a NOXA knockout mutant negatively regulated the growth and development [28]. *FgNOXD* was identified in *Fusarium graminearum*; the NOXD deletion mutant appeared attenuated in growth and conidia, while the sexual development was completely abolished [36]. Studies show that NOX mediates ROS generation and regulated appressorium formation in *Verticillium dahliae* [56]. In the current experiment, the expression levels of *AaNOXA* and *AaNOXB* also increased at an early stage (Figure 9), which showed the uniformity with the change in NOX activities (Figure 7). The theory that NOX activity is positively correlated with ROS accumulation has been demonstrated in *P. expansum* [28], *V. dahliae* [56], and *F. sulphreum* [41]. In our present study, exogenous L-arginine significantly increased the NOX activity and cellular ROS levels in *A. alternata*, and the corresponding gene transcript levels increased. As expected, earlier ROS burst and subsequently enhanced pathogenicity in *A. alternata* were attributed to the effect of exogenous L-arginine.

Excessive ROS accumulated in the plants during the infection stage caused by the pathogen led to oxidative damage to the pathogen, thus delaying the infection process [2]. The ROS scavenging system in fungi (including antioxidant enzymes such as SOD, CAT, POD, GPX and GR) reduced the toxicity of ROS, maintained intracellular oxidative homeostasis, and ultimately promoting infection [21]. Previous studies have shown similar findings linking the activation of antioxidant enzymes to the alleviation of oxidative stress in fungi during the infection process. During infection in *A. alternata*, SOD, CAT, and GPX, activities were increased in response to the ROS burst [29]. In our experiment, a lot of ROS were induced by L-arginine in the early period of inoculation in *A. alternata*. Meanwhile, in the middle and late period of inoculation, low levels of L-arginine induced increased SOD, CAT POD, and GPX activities, and facilitated the scavenging of O_2_^−^ and H_2_O_2_ (Figure 7). L-arginine treatment resulted in a rapid decrease in SOD, CAT, and GPX activities after reaching a peak, which might be related to the enhanced ROS scavenging capacity [57]. This mechanism is related to the upregulation of the expression of genes related to oxidation–reduction reactions at the transcriptional level [57]. It has been reported that exogenous L-arginine increases the activities of SOD and POD in *Agaricus bisporus*, and reduces the accumulation of hydrogen peroxide to protect organisms from oxidative stress [58]. These findings also provide previous evidence for the thoughts presented in the present study.

Furthermore, polyamines possess a unique multi-cationic structure, which gives them the function of protecting cells from the damage caused by ROS. Studies have shown that polyamines can act as an active oxygen scavenger, and activate the antioxidant enzyme system [59,60,61,62]. Spermidine and spermine are shown to be very efficient against alkyl, hydroxyl, and peroxyl radicals [59]. In *U. maydis* ODC mutants, the polyamine mutant is more sensitive to environmental H_2_O_2_ compared to wild-type cells [13]. As shown by Wu et al. [63], putrescine from ODC-mediated generation regulates ROS production to affect secondary metabolism in the basidiomycete *Ganoderma lucidum*. The increase in PAs levels may be accompanied by a decrease in the ROS content [64]. In the current experiment, we also observed the increase in polyamine content in *A. alternata* after L-arginine treatment (Figure 3). This suggests that polyamines may play an important positive role in protecting the cells of *A. alternata* from the toxic effects of ROS.

In plant tissues, most of the cell wall components are polysaccharides, and cell wall-degrading enzymes produced by the pathogen during the infection process are able to degrade the polysaccharides of the plant cell wall, which in turn help the pathogen to infect the host plant [34]. For example, cell wall-degrading enzymes generated by *A. alternata* help to infected plants and cause brown spot disease in citrus [65]. PG and Cx from *Penicillium digitatum* promote the infection of post-harvest citrus fruit [66]. The present results indicate that low levels of L-arginine induce an increase in the cell wall-degrading enzyme activities of *A. alternata* in vitro and in vivo (in kiwifruits inoculated with *A. alternata*), respectively, and the effect may be even more remarkable in vivo (Figure 8). In addition, in the in vivo assay, the PG activity of *A. alternata* increased in the L-arginine treatment group and increased rapidly in the early stage of culture; PMG, PMTE, and PGTE also had a similar trend, whereas the cellulase activity increased rapidly in the late stage (Figure 8). Ramos et al. [67]. reported that PG was first activated in infecting soybean by *Colletotrichum truncatum*, which assisted PMG and Cx in degrading cell wall components of soybeans. Our experiments showed similar results. Therefore, it is cautiously speculated that *A. alternata* first secreted PG to degrade the fruit cell wall in vivo, and the degraded cell wall, while providing a carbon source for *A. alternata*, also facilitated the cellulase degradation of other the components in the fruit cell wall, which ultimately accelerated the infection. Moreover, the significant increase in the *A. alternata* cell wall-degrading enzymes in vivo might be attributed to the combination of the intracellular signal transmission of the NO and ROS and response to the plant defense of *A. alternata* [68].

The above results indicated that lower concentrations of exogenous L-arginine improved the pathogenicity of *A. alternata* by stimulating the accumulation of endogenous arginine polyamines and NO, regulating ROS metabolism, and activating cell wall-degrading enzymes. Therefore, we conservatively hypothesize that L-arginine might be involved in the accumulation of polyamines, NO and ROS metabolism, and that these signaling molecule are involved in the infection process. In addition, further investigations are needed to carry out the underlying mechanism at the molecular level and at the multi-omics level.

## 5. Conclusions

In conclusion, our present study indicated that a lower concentration of L-arginine (5 mM) treatment could promote the pathogenicity of *A. alternata* in kiwifruit. The growth of *A. alternata* was accelerated in vitro and in kiwifruit. The pathogenicity was enhanced by improving the endogenous NO, polyamines, ROS levels, and the induced activities and gene expression levels of the cell wall-degrading enzymes. In addition, the activated antioxidant system, including SOD, CAT, POD, GR, and GPX, maintained a redox balance during the infection, which finally contributed to the promotion of *A. alternata* pathogenicity in kiwifruit. However, excessive concentrations (7.5 and 10 mM L-arginine) showed an opposite effect on pathogenicity, probably due to the structural damage of *A. alternata*. This study innovatively revealed the mechanism by which low concentrations of L-arginine increase the pathogenicity of *A. alternata*, and also may provide a theoretical basis for the specific and precise targeting of *A. alternata* in kiwifruit. However, given that pathogenicity is likely to be regulated by other potential factors, further studies at the molecular level are needed for better comprehension.

## Figures and Tables

**Table 1:** Primer sequences used for real-time quantitative PCR (RT-qPCR).

Gene	Forward Primer (5′-3′)	Reverse Primer (5′-3′)	Product Size (bp)
Actin	TACACTTTCTCAACCACAGCCG	CGGAATCGCTCGTTACCAAT	176
NOXA	GGACCCACTCACCGAACTCAAATC	CCATCTCGCATACCGCAGAACAG	81
NOXB	GTGCTGCCCTGAAATCTCCATCTG	CTTCCTCTCCGTGCTACAACCAAG	148
SOD	GGAGCAAAGGCTGTCTATCGT	TTGCCGTTCTGGTATTGGAG	123
CAT	AGTCGGAGGAGCAAATCACAG	AGTCGGAGGAGCAAATCACAG	266
POD	TTAACTACGGCGTTAGCTTCC	TTAACTACGGCGTTAGCTTCC	228
GPX	TTAACTACGGCGTTAGCTTCC	TTAACTACGGCGTTAGCTTCC	202
GR	GTGGAGCCAATCCCAGAAA	GTGGAGCCAATCCCAGAAA	217
Cx	CACCTCGCTCGCTCCTTTCC	CCATATCCAGCAGGCTCAACATTG	132
β-1,3-glucanase	CGGCAATGCTCCAGGTTAT	CGCACGATAACATAGAAAGGAA	196
PG	CTCACAAACTGACCGACTCCA	CATCGCAGCCGTTGATACTA	84
PMTE	CAGAAGTGGAACGGTGACAACAAC	TGATAGGCACAGGCTTCGCAAG	127

## Data Availability

Data will be made available on request.
